# Therapeutic Potential of a Combination of Electroacupuncture and Human iPSC-Derived Small Extracellular Vesicles for Ischemic Stroke

**DOI:** 10.3390/cells11050820

**Published:** 2022-02-26

**Authors:** Peiying Deng, Liang Wang, Qiongqiong Zhang, Suhui Chen, Yamin Zhang, Hong Xu, Hui Chen, Yi Xu, Wei He, Jianmin Zhang, Hua Sun

**Affiliations:** 1 Department of Traditional Chinese Medicine, Peking Union Medical College Hospital, Peking Union Medical College, Chinese Academy of Medical Sciences, Beijing 100730, China; dengpeiying379@outlook.com (P.D.); qiongqzhang@163.com (Q.Z.); suhui520220@sina.com (S.C.); yaminzhanghn@163.com (Y.Z.); xuhong10850@163.com (H.X.); 2 CAMS Key Laboratory for T Cell and Immunotherapy, State Key Laboratory of Medical Molecular Biology, Department of Immunology, Institute of Basic Medical Sciences, Chinese Academy of Medical Sciences and School of Basic Medicine, Peking Union Medical College, Beijing 100005, China; liang.wang@ibms.pumc.edu.cn (L.W.); chenhui_1980@126.com (H.C.); xuyi2207@163.com (Y.X.); 3 Changzhou Xitaihu Institute for Frontier Technology of Cell Therapy, Changzhou 213000, China; 4 Guidon Pharmaceutics, Beijing 100176, China

**Keywords:** electroacupuncture, induced pluripotent stem cell, ischemic stroke, inflammation, IL-33/ST2

## Abstract

This paper aimed to explore the roles of the combination of electroacupuncture (EA) and induced pluripotent stem cell-derived small extracellular vesicles (iPSC-EVs) on mice with ischemic stroke and the underlying mechanisms. A focal cerebral ischemia model was established in C57BL/6 mice through middle cerebral artery occlusion (MCAO). After 3 days, neurological impairment and motor function were examined by performing behavioral tests. The infarct volume and neuronal apoptosis were examined using TTC staining and TUNEL assays. Flow cytometry was performed to assess the proliferation of T lymphocytes. The changes in the interleukin (IL)-33/ST2 axis were evaluated by immunofluorescence and Western blotting. The combination of EA and iPSC-EVs treatment ameliorated neurological impairments and reduced the infarct volume and neuronal apoptosis in MCAO mice. EA plus iPSC-EVs suppressed T helper (Th1) and Th17 responses and promoted the regulatory T cell (Treg) response. In addition, EA plus iPSC-EVs exerted neuroprotective effects by regulating the IL-33/ST2 axis and inhibiting the microglia and astrocyte activation. Taken together, the study shows that EA and iPSC-EVs exerted a synergistic neuroprotective effect in MCAO mice, and this treatment may represent a novel potent therapy for ischemic stroke and damage to other tissues.

## 1. Introduction

Ischemic stroke, a common neurological disorder, leads to permanent disability in patients worldwide [1,2,3]. Neuroinflammation induced by ischemic stroke plays an essential role in multiple mechanisms leading to secondary brain injury after stroke [4]. The current clinical optimal therapy is intravenous thrombolysis or endovascular thrombectomy, but both are time-critical procedures [5,6,7,8,9,10]. Hence, the development of a new therapeutic approach is imperative to reduce ischemia-reperfusion injury.

T lymphocytes are key mediators of neuroinflammation induced by ischemic stroke [11]. T helper 1 cells (Th1) promote inflammation, and Th2 cells preferentially induce anti-inflammatory responses [12]. Therefore, the Th1/Th2 balance may provide insight into the regulation of the inflammatory response. Moreover, a previous study showed a significant increment of Th17 cells and a marked reduction of peripheral regulatory T (Treg) cells in ischemic stroke patients, suggesting that the peripheral Th17/Treg imbalance might underlie the pathogenesis of ischemic stroke [13]. Recent studies have also reported an upregulation of Treg cell numbers in the mice brain, and these cells may promote functional recovery and brain repair following stroke [14,15]. Thus, a deeper study of the effects of T lymphocytes is imperative for ischemic stroke therapy.

Interleukin (IL)-33, from the IL-1 cytokines family, is widely expressed in brains and spinal cords of human and rodent [16,17]. Emerging evidence increasingly implicates a pleiotropic role of IL-33 on regulating T lymphocyte immune responses [18,19,20]. IL-33 functions both as a transcription factor upon an interaction with chromosomes and as a traditional cytokine released from necrotic cells, acting as alarmin to alert the body to tissue injury [17,21]. IL-33 as a promoter is involved in the pathological progression of Th2-related diseases by binding to its receptor ST2 selectively expressed on Th2 cells [22,23]. Recent studies also supported the positive effects of IL-33 on several diseases by shifting the Th1/Th2 balance toward Th2 [24,25,26]. Nevertheless, the functions of IL-33 in ischemic stroke remain disputable. In ischemic stroke patients, IL-33 plasma level was found to be significantly elevated [27]. A recent study indicated that IL-33 ameliorates ischemic damage by encouraging the Th2 response and inhibiting the Th17 response in mice [28]. Moreover, the endogenous IL-33/ST2 axis might encourage beneficial responses of microglia and attenuate ischemic brain damage after stroke [29].

Induced pluripotent stem cells (iPSCs) have emerged as one of the most promising sources of stem cells for cerebrovascular disease therapy since iPSC technology was discovered in 2006 [30], as they possess tremendous advantages for tissue regeneration, self-renewal capacity, and differentiation potential. Although iPSC administration is restricted by a low engraftment rate and the potential tumorigenesis risk, recent research has proven that the advantageous effects of transplanted stem cells are largely attributable to exosomes released from them [31,32,33], as exosomes have low immunogenicity, low toxicity, and the capacity to cross the blood-brain barrier [34,35]. The administration of exosomes has been used as a therapeutic approach for ischemic stroke. Exosomes secreted by M2 microglia have been reported to ameliorate ischemic brain damage and promote neuronal survival [36]. A study also reported that mesenchymal stem cell (MSC)-derived exosomes are beneficial for neuronal remodeling and functional recovery after stroke [37]. Based on accumulating evidence, exosomes have improved the functional outcomes of ischemic stroke therapy through their cargoes, including lipids, proteins, and nucleic acids [35,38]. A recent report confirmed that exosomes derived from iPSC-generated neurons facilitate neurogenesis and regulate neural circuit development [39]. These observations suggest that the application of iPSC-derived small extracellular vesicles (iPSC-EVs) may be a promising method for stroke therapy; however, their cellular targets remain poorly defined.

Electroacupuncture (EA), as a technique combining traditional acupuncture and electrical stimulation, has been used for stroke and poststroke rehabilitation, as it ameliorates neurological impairments with no obvious adverse effects. Research has indicated that EA treatment effectively attenuates inflammatory injury and exerts a neuroprotective effect in ischemic stroke. Thus, both EA and iPSC-EVs are promising clinical treatment strategies for stroke. We reported that EA treatment at “Baihui” (GV20) and “Zusanli” (ST36) acupoints could alleviate neuronal injury in middle cerebral artery occlusion (MCAO) rats [40,41]. However, the specific mechanism by which EA and iPSC-EVs regulate the neuroinflammation remains unclear.

Accordingly, the current study explored the potential effects of the combination of EA treatment and iPSC-EVs on cerebral ischemic injury and found that EA and iPSC-EVs regulated the IL-33/ST2-induced inflammatory response in MCAO mice.

## 2. Materials and Methods

### 2.1. Animals

Male C57BL/6 mice aged 6–8 weeks (*n* = 10 per group) were purchased from Beijing Vital River Laboratory Animal Technology Co., Ltd. (Beijing, China). The mice were housed at 22 ± 2 °C under alternating 12 h light/dark cycles. All experimental protocols were conducted according to the guidelines of the National Institutes for Animal Research and approved by the Ethics Committee for Animal Experimentation of Peking Union Medical College Hospital of the Chinese Academy of Medical Sciences (reference no. XHDW-2019-056).

### 2.2. Mouse Model of MCAO

Transient focal cerebral ischemia was established by the right MCAO in mice for 1 h [29]. Briefly, animal models were performed on by inserting a silicon rubber-coated 7-0 monofilament (Doccol Corporation, Sharon, MA, USA) via the right external carotid artery (ECA) across the right internal carotid artery (ICA) to the origin of the MCA. Reperfusion was established by withdrawing the monofilament. Regional cerebral blood flow (rCBF) was supervised with a laser speckle contrast imager (PeriCam PSI HR System, Jarfalla-Stockholm, Sweden). Animals that died or failed to show a ≥80% rCBF reduction were excluded from further analysis. The same procedure was performed for sham group mice but without MCAO. Mice were housed individually after the operation and euthanized after 3 days for further experiments. The model success rate was 93.33% and the mortality rate was 6.66%.

### 2.3. Isolation and Characterization of iPSC-EVs

The iPSCs (GD01-009) were gifted from Guidon Pharmaceutics (Beijing, China) and maintained in mTeSR1 plus (100-0276, Stemcell Technologies, Vancouver, BC, Canada) on Matrigel-coated plates (354277, Corning, NY, USA). The iPSCs were passaged as clumps using Versene Solution (15040066, Thermo Fisher Scientific, Watham, MA, USA) at a ratio of 1:4–1:6 every 4–5 days. The culture medium was substituted daily with GDEV medium (GDSJ0040, Guidon Pharmaceutics, Beijing, China) and centrifuged after daily collection to remove larger particles [42]. The supernatant was filtered and concentrated with Amicon Ultra-15 Centrifugal Filters (Ultracel-100 kDa, Merck Millipore, Burlington, MA, USA). The collected iPSC-EVs were stored at −80 °C.

The diameter, particle number and surface markers (CD9 or CD63) of iPSC-EVs were analyzed using a Flow NanoAnalyzer (NanoFCM, Xiamen, China). Transmission electron microscopy was used to identify the morphology of iPSC-EVs.

### 2.4. iPSC-EVs, EA and Sham Acupuncture Treatment

For iPSC-EVs treatment, each mouse received 20 μg each injection with a total of three injections at 2, 24, and 48 h after MCAO surgery. EA or sham acupuncture treatment was performed three times at 0 h, 24 h and 48 h, respectively after reperfusion. For the EA acupuncture group, acupuncture needles (Zhongyan Taihe, Beijing, China) were stuck into the mice at “Baihui” acupoint (GV20, on the midline of the head and the line connecting the apices of both auricles) and left “Zusanli” acupoint (ST36, 5 mm distal to the head of the fibula beneath the stifle and 2 mm lateral to the tibial tuberosity) at a depth of 2–3 mm. EA stimulation was carried out for 30 min at 2 Hz (intensity, 1 mA) frequency with continuous waves using an electroacupuncture device (KWD-808 II, Great Wall Brand, Baoding, China). For sham acupuncture treatment, 10 mm above the anterior superior iliac spine was selected as the insertion site but without EA stimulation.

### 2.5. Neurological Deficit Assessment

Clark focal neurological score shows a strong correlation with infarct volume, which was used to evaluate the neurological deficit. The test provides a total score from 0 to 28 points. Animals with normal function scored the lowest (0 points), whereas the highest score (28 points) represented the most severe functional impairment.

### 2.6. Behavioral Tests

The forelimb functional recovery of mice was assessed using the cylinder test. Briefly, mice were positioned in a glass cylinder and moved freely for 10 min during video recording. A higher percentage of contralateral (left) limb use means better forelimb functional recovery.

Open field test was also conducted to estimate the mobility of mice. All mice were tested separately in an open field apparatus consisting of a black square arena (50 cm × 50 cm) with black walls (60 cm) for 5 min. The following parameters were obtained and analyzed using Labmaze V3.0 software: total distance traveled (cm), moving time (s) and velocity (cm/s).

### 2.7. Infarct Volume Measurement

All animals were anesthetized and sacrificed at 72 h after reperfusion. Brain sections collected from separate brains of each group (*n* = 5 per group) were incubated using 2% TTC (G3005, Solarbio, Beijing, China) and fixed with 4% paraformaldehyde overnight (Figure S1). Sections were scanned and analyzed with ImageJ software. The infarct volume (%) was calculated as follows: (contralateral hemispheric volume—undamaged ipsilateral hemispheric volume)/contralateral hemispheric volume × 100%.

### 2.8. Flow Cytometry

Spleens of mice were digested and passed using a 40 μm nylon mesh. Then, peripheral blood was collected and suspended in PBS. Splenocytes were stimulated with a cell stimulation cocktail (00-4975-93, eBioscience, San Diego, CA, USA) and Th cells subtypes were identified using the following antibodies (BioLegend, San Diego, CA, USA): anti-CD3-FITC (100204), anti-CD4-Brilliant Violet 650™ (100545), anti-IFN-γ-APC (505810), anti-IL-4-PE-Cy7 (504118), anti-IL-17A-Brilliant Violet 421™ (506926) and anti-Foxp3-PE (320008). Flow cytometry was carried out with a FACSCalibur cytometer and data were analyzed with CellQuest software (Beckman Coulter, Brea, CA, USA).

### 2.9. Immunofluorescence Staining

Frozen brain sections (12 μm thick) were incubated with rabbit anti-NeuN (ab177487, Abcam, Cambridge, MA, USA), goat anti-IL-33 (AF3626, R&D Systems, Minneapolis, MN, USA), rabbit anti-SULT2A1/ST2 (ab194113, Abcam, Cambridge, MA, USA), goat anti-GFAP (ab68428, Abcam, Cambridge, MA, USA), and rabbit anti-Iba1 (019-019741, Wako, Japan). Stained sections were incubated with fluorescent secondary antibody and mounted for nuclear labeling using fluorescence mounting medium containing DAPI. We used Roche In Situ Cell Death Detection Kit (11684817910) to perform TUNEL staining (fluorescein). After nuclear staining, the slices were visualized and imaged with a fluorescence microscope. Images were captured at ×20 magnification. The numbers of IL-33^+^, ST2^+^, GFAP^+^ and Iba1^+^ cells were counted and normalized to the area. Forty-five images (3 field/section, 3 sections/animal, *n* = 5) of each group were analyzed using ZEN software.

### 2.10. Western Blot

Mouse brain tissue around the penumbra area was collected from each group. The samples of Western blotting and immunofluorescence were collected from the same fresh brain by splitting at the middle of infarct area into two parts. Protein samples were separated on SDS-PAGE gels and electrotransferred to nitrocellulose membranes (Millipore, Burlington, MA, USA). The membranes were hybridized with the primary antibodies, including goat anti-IL-33 antibody (1:2500, AF3626, R&D Systems, Minneapolis, MN, USA) and rabbit anti-SULT2A1/ST2 antibody (1:1000, ab194113, Abcam, Cambridge, MA, USA), followed by incubation with secondary antibodies. The protein bands were scanned and analyzed with ImageJ software. All bands were normalized to β-actin (ab8227, Abcam, Cambridge, MA, USA).

### 2.11. Statistical Analysis

All data are expressed as the means ± standard errors of the means (SEM) and analyzed using SPSS 26.0 software. One-way analysis of variance (ANOVA) with the Tukey–Kramer post hoc multiple comparisons test were used to identify significant differences among groups (*p* < 0.05).

## 3. Results

### 3.1. Combined Therapy with EA and iPSC-EVs Ameliorates Motor Dysfunction after Ischemic Stroke

**Figure 1:**
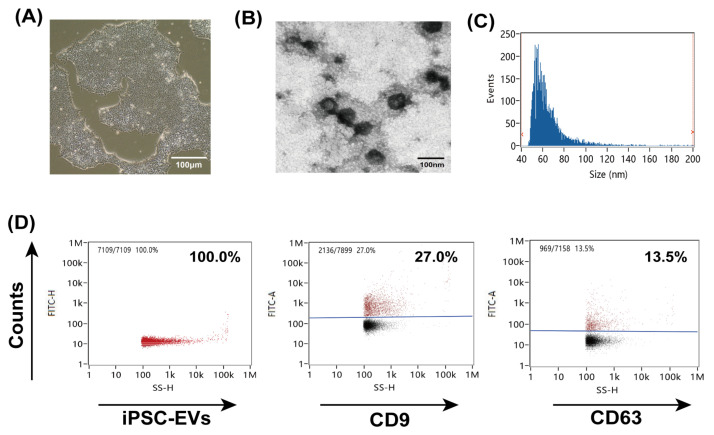
Characterization of human iPSC-EVs. (**A**) Typical image of human iPSCs cultured in vitro. Scale bar = 100 μm. (**B**) TEM examination of the morphology of iPSC-EVs. Scale bar = 100 nm. (**C**,**D**) Nanoflow cytometry was used to detect the size and surface markers CD9 and CD63 of iPSC-EVs.

**Figure 2:**
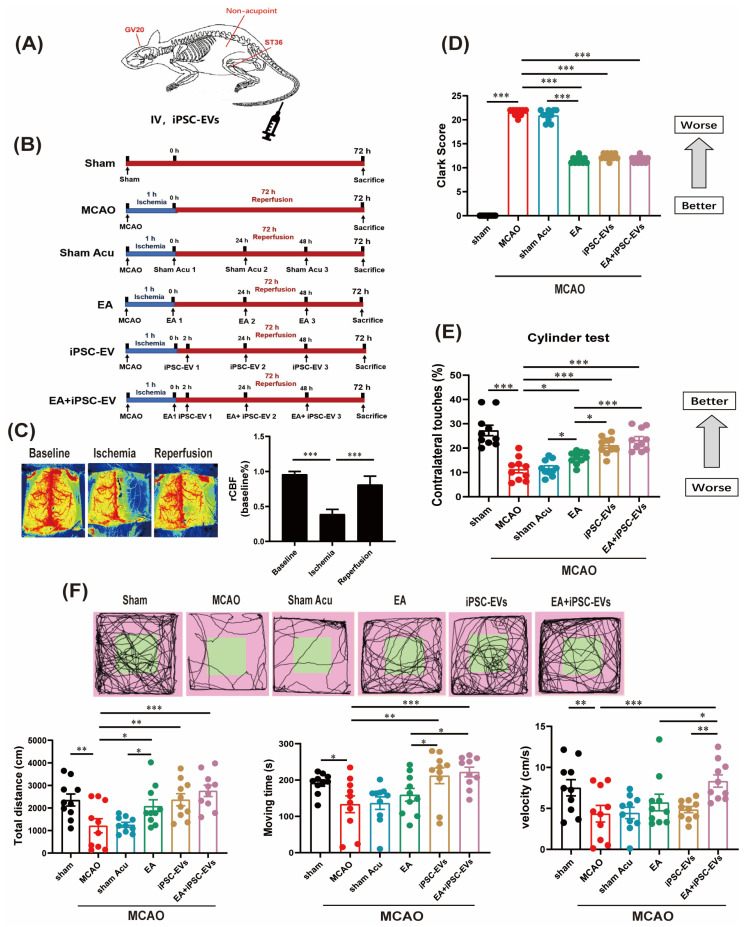
EA stimulation combined with iPSC-EVs improves motor function after ischemic stroke. (**A**) Map of acupoint and nonacupoint locations in mice. (**B**) Timeline of the experimental design in different groups. (**C**) Quantification of rCBF monitored using laser speckle imaging before and after MCAO, as well as 5 min after reperfusion. (**D**) Neurological deficits were evaluated by calculating the Clark score. (**E**,**F**) Cylinder test and open field test were used to assess the deficits in motor function of MCAO mice. Data are shown as means ± SEM. * *p* < 0.05, ** *p* < 0.01, and *** *p* < 0.001.

iPSC culture medium was harvested when the cells reached 80–90% confluence (Figure 1A), then iPSC-EVs were purified by ultracentrifugation using Amicon Ultra-15 or Centricon Plus-70 Centrifugal Filter Units (Millipore). TEM was applied to observe the morphology of iPSC-EVs (Figure 1B). Nanoflow cytometry was used to examine the size and expression of the surface markers CD9 and CD63 in iPSC-EVs (Figure 1C,D). We used a MCAO mouse model to determine the effects of EA and iPSC-EVs on the motor function of mice with ischemic stroke. Detailed acupoint locations are shown in Figure 2A. The mice were classified into six groups, as shown in Figure 2B. rCBF was monitored before and during ischemia, as well as 5 min after reperfusion. The rCBF reduction was stable throughout the occlusion period and recovered to preischemic levels immediately upon removal of the filament in all groups (Figure 2C). First, the neurological impairment of mice was assessed using a focal neurological scale. The Clark score was significantly higher in the MCAO group than that in sham group. After treatment with EA, iPSC-EVs or both, the Clark scores were substantially reduced, indicating a significant alleviation of the neurological impairment (Figure 2D). Sham acupuncture treatment did not change the score after MCAO (Figure 2D). Both the cylinder (Figure 2E) and open field (Figure 2F) tests also showed that the mice in the EA, iPSC-EVs and EA+iPSC-EVs groups exhibited a significant improvement in movement at 72 h after MCAO. In particular, the EA+iPSC-EVs group showed a more significant improvement (Figure 2F), indicating that EA combined with iPSC-EVs treatment exerted a better effect on improving neurological performance and motor function after ischemic brain injury.

### 3.2. EA and iPSC-EVs Treatment Attenuates Ischemic Brain Damage

**Figure 3:**
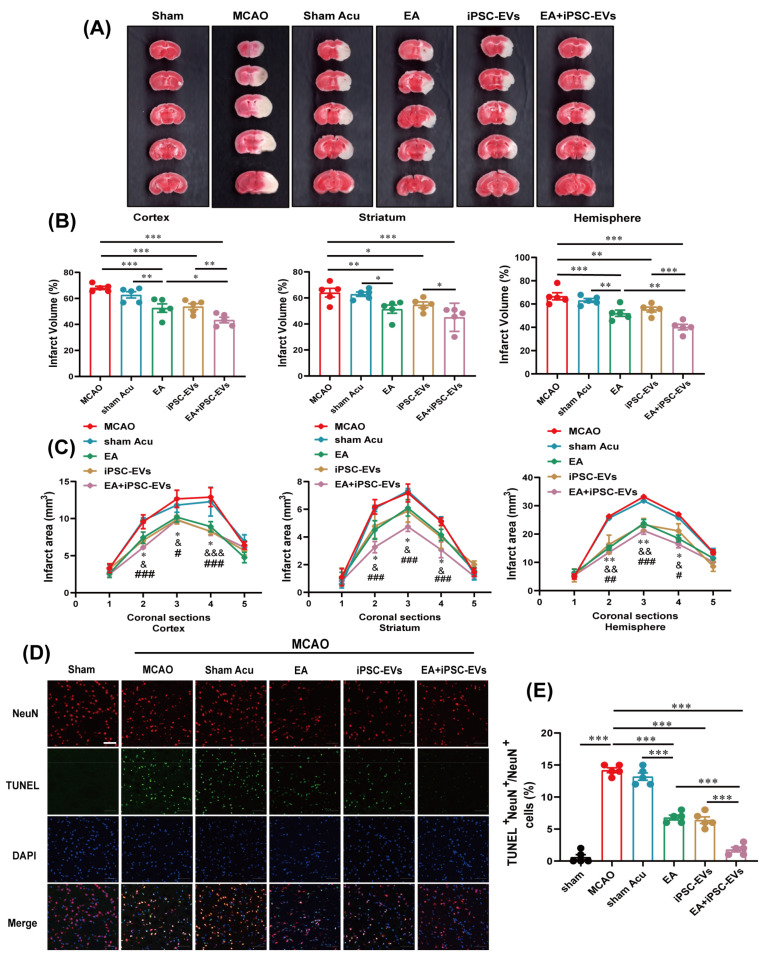
EA and iPSC-EVs treatment attenuates ischemic brain damage in MCAO mice. (**A**) Representative images of TTC staining at 72 h in MCAO mice. (**B**) Quantification of infarct volumes in the cortex, striatum and whole hemisphere of MCAO mice. (**C**) Quantification of the infarct area among the five coronal levels (level 1 is most anterior) in MCAO mice. (**D**) Representative images of NeuN and TUNEL staining in the infarct area of MCAO mice at 72 h. Scale bar = 50 μm. (**E**) Quantification of NeuN and TUNEL double positive cells. Data are shown as means ± SEM. * *p* < 0.05, ** *p* < 0.01, and *** *p* < 0.001 for the EA group vs. the model group; ^&^ *p* < 0.05, ^&&^ *p* < 0.01, and ^&&&^ *p* < 0.001 for the iPSC-EVs group vs. the model group; ^#^ *p* < 0.05, ^##^ *p* < 0.01, and ^###^ *p* < 0.001 for the EA+iPSC-EVs group vs. the model group.

To confirm the effects of EA and iPSC-EVs in MCAO mice, the infarct volumes were examined with TTC staining and neuronal apoptosis was analyzed with immunofluorescence. In the MCAO group, ischemia-induced infarct volumes were significantly larger at 72 h after stroke (Figure 3A). Surprisingly, treatment with EA and iPSC-EVs markedly decreased the ischemia-induced infarct volume, while treatment with sham acupuncture had no effect on the infarct volume. In addition, compared with MCAO mice, the infarct volume in EA-treated mice was reduced by 15.5% in the cortex, 12.6% in the striatum and 14.5% in the entire hemisphere, while in iPSC-EVs-treated and EA+iPSC-EVs-treated mice, the infarct volume was reduced by 14.4% and 24.7% in the cortex, 9.3% and 18.9% in the striatum, and 11.4% and 26.5% in the entire hemisphere, respectively (Figure 3B). Moreover, the reduction in infarct size was not skewed to a particular level (Figure 3C). Furthermore, the results from the TUNEL assay confirmed a significant increase in neuronal apoptosis in the infarct core area of MCAO mice compared with sham mice, and the magnitude of neuronal apoptosis was significantly lower in mice treated with EA+iPSC-EVs (Figure 3D). There was no significant difference between the EA and iPSC-EVs groups, but significant differences were observed between the EA and sham acupuncture groups (Figure 3E). Taken together, EA or iPSC-EVs attenuate ischemia-induced cerebral injury in a mouse model of MCAO and the overall therapeutic effect of EA combined with iPSC-EVs is superior to that of the other two agents.

### 3.3. EA and iPSC-EVs Treatment Modulates the Proliferation of Th Cells in MCAO Mice

**Figure 4:**
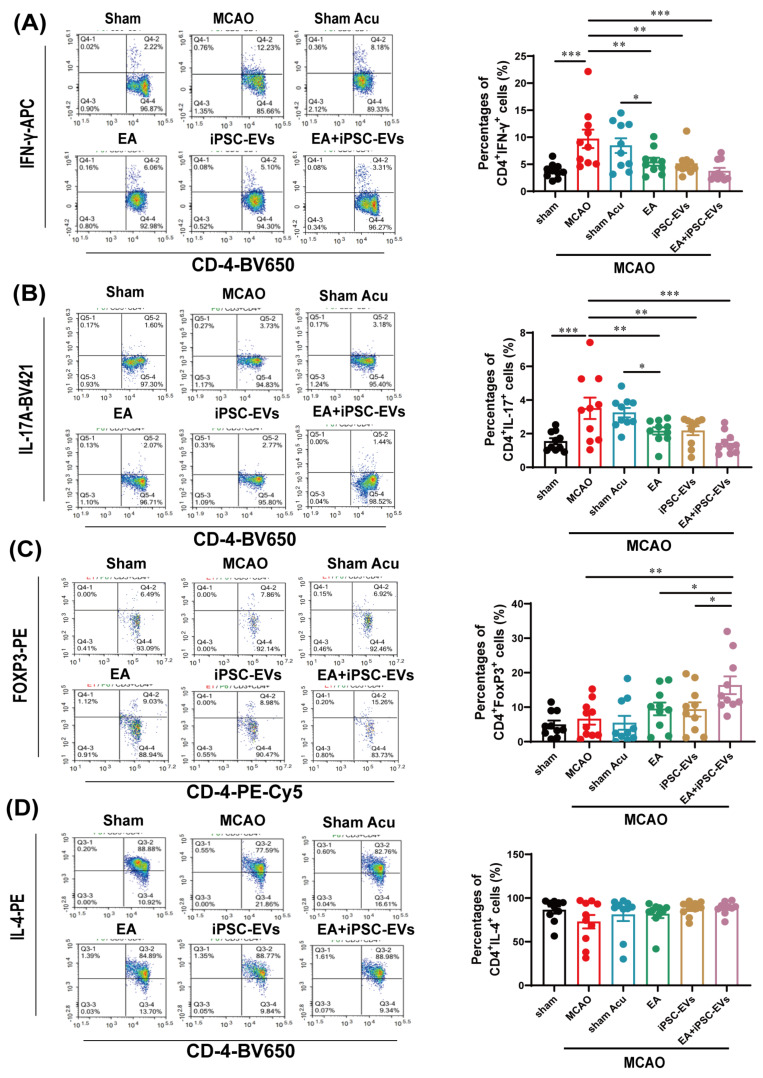
EA and iPSC-EVs treatment modulates the proliferation of Th cells. (**A**) The level of IFNγ^+^ Th cells in mice after stroke. (**B**) The level of IL-17^+^ Th cells in mice after stroke. (**C**) The level of Foxp3^+^ Th cells in mice after stroke. (**D**) The level of IL-4^+^ Th cells in mice after stroke. Data are shown as means ± SEM. * *p* < 0.05, ** *p* < 0.01, and *** *p* < 0.001.

We investigated the expression of Th cell subsets (namely, IFN-γ^+^ Th cells, IL-4^+^ Th cells, IL-17^+^ Th cells and Foxp3^+^ Treg cells) in the peripheral blood of mice 72 h after MCAO using flow cytometry to explore whether EA or iPSC-EVs modulated MCAO-induced T cell immune responses. The levels of IFN-γ^+^ Th cells (Figure 4A) and IL-17^+^ Th cells (Figure 4B) were significantly increased after MCAO, whereas these abnormal increases were significantly suppressed in the EA-, iPSC-EVs- or EA+iPSC-EVs-treated animals, although the reduction was significantly greater in the EA+iPSC-EVs group. There was no significant difference among the three treatment groups, but significant differences were observed between the EA and sham acupuncture groups. In contrast, the combination of EA and iPSC-EVs exerted a significant synergetic effect on preventing the MCAO-induced reduction in the quantity of Foxp3^+^ Treg cells (Figure 4C). The number of IL-4^+^ Th cells did not differ significantly among any of the groups after MCAO (Figure 4D). Based on these results, the combination of EA and iPSC-EVs treatment is a more efficient therapeutic approach to modulate MCAO-induced inflammatory responses.

### 3.4. EA and iPSC-EVs Treatment Modulates the IL-33/ST2 Activation of in Astrocytes

**Figure 5:**
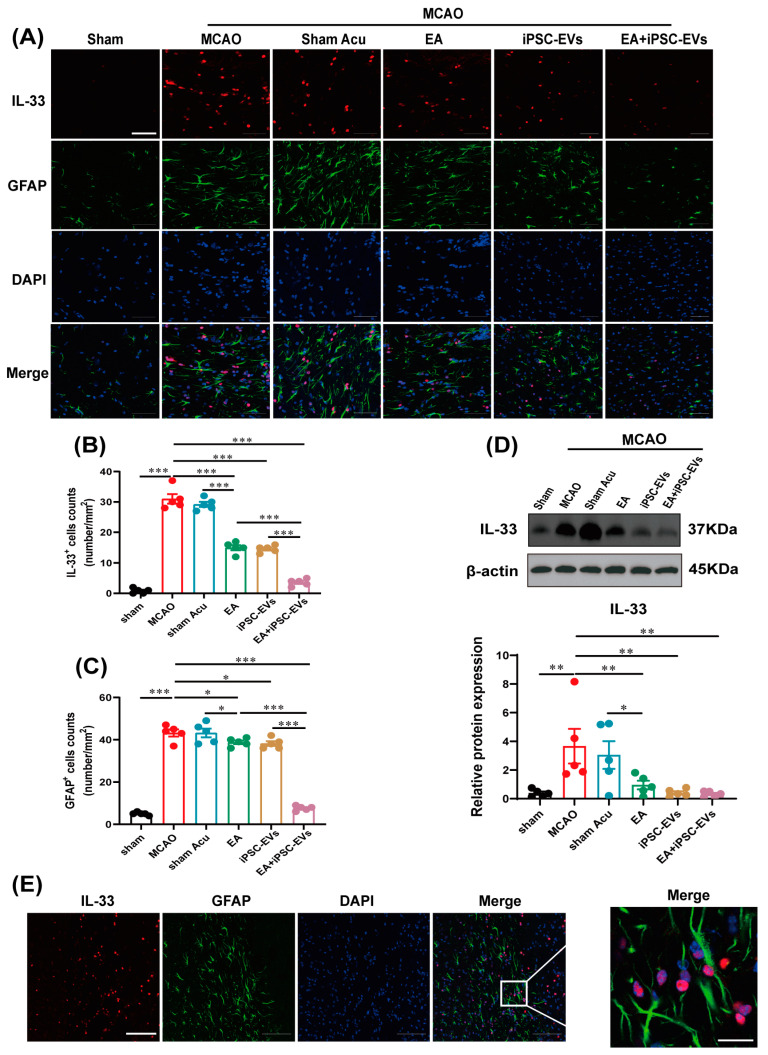
EA and iPSC-EVs treatment reduces IL-33 expression in astrocytes after ischemic stroke. (**A**) Representative images of IL-33 (red) and GFAP (green) labeling in the brains of MCAO mice at 72 h. Scale bar = 50 μm. (**B**) The number of IL-33^+^ cells in MCAO mice. (**C**) The number of GFAP^+^ cells in MCAO mice. (**D**) Western blot analysis of IL-33 levels in the brains of MCAO mice. (**E**) Colocalization of IL-33 in GFAP^+^ cells. Scale bar = 100 μm in the first four images and 20 μm in the last image of this panel. Data are shown as means ± SEM. * *p* < 0.05, ** *p* < 0.01, and *** *p* < 0.001.

**Figure 6:**
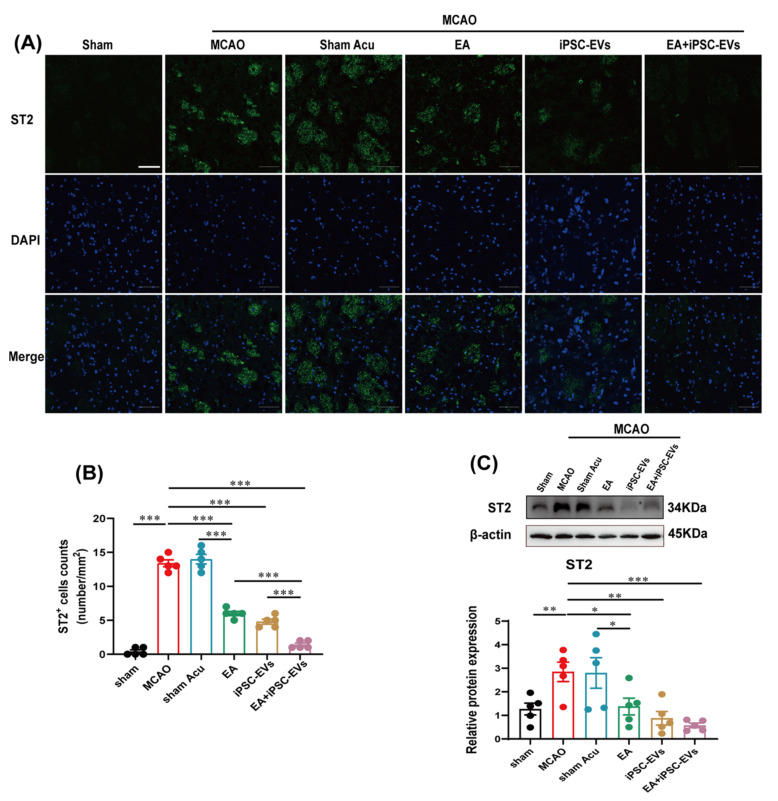
Effects of EA and iPSC-EVs treatment on ST2 expression in MCAO mice. (**A**) Representative images of ST2 (green) labeling in the brains of MCAO mice at 72 h. Scale bar = 50 μm. (**B**) The level of ST2^+^ cells in MCAO mice. (**C**) ST2 levels in the brains of MCAO mice were detected using Western blotting. Data are shown as means ± SEM. * *p* < 0.05, ** *p* < 0.01, and *** *p* < 0.001.

IL-33 is involved in regulating neuroinflammation by binding to the ST2 receptor on the surface of astrocytes or microglia [16]. Immunofluorescence and Western blotting were carried out to assess the contribution of EA and iPSC-EVs on the activation of IL-33/ST2. Both the number of IL-33 immunoreactive cells (Figure 5A–B) and the level of the IL-33 protein (Figure 5D) were significantly increased at the lesion site in MCAO mice compared to sham control mice. Compared with EA or iPSC-EVs treatment alone, treatment with EA+iPSC-EVs exerted a better inhibitory effect on the MCAO-induced increase in IL-33 expression. Although EA and iPSC-EVs treatment markedly inhibited the MCAO-induced upregulation of IL-33 in the infarct area at 72 h, no significant difference was observed between these treatments. Consistently, GFAP staining showed that MCAO-induced IL-33 expression in the brain was accompanied by activation of astrocytes in MCAO mice, and this change was significantly alleviated by treatment with EA, iPSC-EVs or EA+iPSC-EVs. This effect was more significant in EA+iPSC-EVs-treated mice, while mice that received sham acupuncture treatment showed no difference from MCAO mice (Figure 5C). Additionally, the immunofluorescence results reflected that IL-33 was localized in astrocytes (Figure 5E). The trend for ST2 expression was similar (Figure 6A–C), as increased ST2 expression in the lesion site at 72 h after MCAO was reversed by EA and iPSC-EVs treatment, especially in the EA+iPSC-EVs-treated mice; sham acupuncture treatment did not have that effect. These findings suggest that EA combined with iPSC-EVs treatment modulates the T cell response by promoting IL-33/ST2 axis activation in astrocytes.

### 3.5. EA and iPSC-EVs Treatment Protects against the Microglia Activation Induced by Cerebral Ischemia

**Figure 7:**
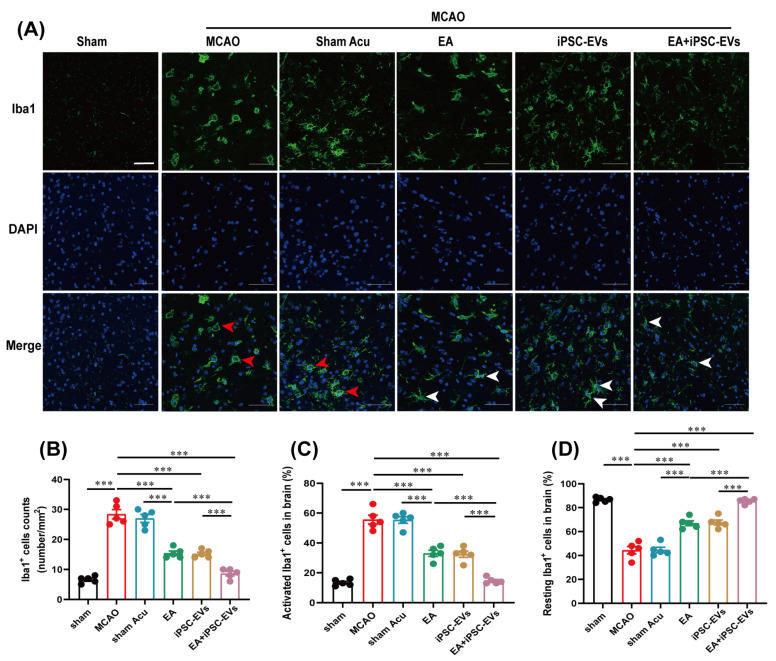
EA and iPSC-EVs treatment modulates microglial activation in MCAO mice. (**A**) Representative images of Iba1 (green) labeling in the brains of MCAO mice at 72 h. The red arrows indicate activated microglia, and the white arrows indicate resting microglia. Scale bar = 50 μm. (**B**) The total number of Iba1^+^ cells in MCAO mice. (**C**) The quantity of activated Iba1^+^ cells in MCAO mice. (**D**) The quantity of resting Iba1^+^ cells in MCAO mice. Data are shown as means ± SEM. *** *p* < 0.001.

A recent study has confirmed a series of changes in the morphology and function of microglia after cerebral ischemia-reperfusion injury [43]. Microglia exist in their “resting” state characterized by a ramified morphology [44]. Activated microglia undergo significant changes in morphology. After ischemia-reperfusion, microglia proliferate and activate rapidly in the infarct region, the perikarya of activated microglia gradually become larger, and their branches decrease [45]. We applied immunofluorescence staining to detect microglial phenotypic markers at 72 h after MCAO and to verify whether EA and iPSC-EVs also inhibited the microglia activation. MCAO induced significant increases in the numbers of total and activated Iba1^+^ cells, whereas in EA-, iPSC-EVs- and EA+iPSC-EVs-treated mice, this increase was significantly suppressed, especially in mice receiving the EA+iPSC-EVs combination treatment (Figure 7A–C). Conversely, a significant decrease in the quantity of resting Iba1^+^ cells was observed after MCAO, and this trend was significantly prevented by EA or iPSC-EVs treatment, especially in the EA+iPSC-EVs group (Figure 7D). Therefore, EA and iPSC-EVs treatment also suppressed the MCAO-induced activation of microglia.

## 4. Discussion

Ischemic stroke promotes increased reactive oxygen species (ROS) and oxidative stress, resulting in the pro-inflammatory cytokines production [46]. Neuroinflammation is a major consequence of focal cerebral ischemia and contributes to neuronal injury. In current research, we studied the neuroprotective effects of iPSC-EVs and EA and explored the possible mechanisms using a MCAO mouse model. Treatment with iPSC-EVs, EA, or a combination of the two substantially alleviated ischemia-induced brain damage. The therapeutic effect of iPSC-EVs and EA was associated with the inhibition of inflammation by regulating the IL-33/ST2 pathway.

Cell-based therapy using iPSCs is a potentially promising approach to alleviate ischemic brain injury. However, the use of iPSCs may be compromised by their tumorigenic potential. According to recent studies, the positive contribution of stem cells is predominantly through its paracrine mechanism, and EVs are pivotal in this process [33,47]. Stem cell-derived exosome technologies have achieved great progress in several fields [35,48]. The therapeutic advantages of iPSC-EVs have been proven in diverse diseases, such as hepatic ischemia-reperfusion injury and acute myocardial ischemia-reperfusion, and these studies have documented encouraging outcomes [31,49]. However, the mechanism of iPSC-EVs for ischemic stroke requires more research.

As an economic and convenient therapeutic method, EA can be applied to relieve symptoms of ischemic stroke. Because of the complexity of the disease, combinations of acupuncture points increased the efficacy of EA compared with single acupuncture points. As shown in our previous studies, EA at the combination of the “Baihui” (GV20) and “Zusanli” (ST36) acupoints exerts synergistic protective effects on attenuating neuronal injury by suppressing endoplasmic reticulum stress and ameliorating mitochondrial functional damage in MCAO rats [40,50]. We demonstrated that the combination of two acupoints provided a significant protection against cerebral ischemic injury within seven days [51], especially on day 3. Thus, in this paper we focused on evaluating the roles of EA and iPSC-EVs treatment on MCAO animals that received treatments for 3 days. Traditional Chinese Medicine theory indicates that GV20 in the head is closely related to the brain and is often applied to treat brain diseases. Another acupoint, ST36, is thought to enrich the body’s energy. The effectiveness of GV20+ST36 might be mainly attributed to the combination of local and distant effects of the acupuncture points. Research shows that stimulation at ST36 leads to different electroencephalography and functional magnetic resonance imaging (fMRI) patterns than the needling of two non-acupuncture points, and brain fMRI provided evidence for acupoint specificity [52]. The current research offers molecular proof for acupoint specificity.

In this research, we injected iPSC-EVs into the tail vein of mice and investigated their damage repair effect on the brain in mice with or without EA stimulation. Both iPSC-EVs alone and EA alone reduced the infarct volume (TTC staining), enhanced neurological performance (Clark score), improved motor function (cylinder test and open field test) and suppressed neuronal apoptosis (TUNEL assay) in MCAO mice. Furthermore, a combination of iPSC-EVs and EA works best. We believe that the combination of EA with iPSC-EVs may be a more effective approach to repair brain damage compared to iPSC-EVs or EA alone. Compared with previous studies, the combination of EA and iPSC-EVs has a synergistic neuroprotective effect against ischemic injury. Notably, the selected acupoints vary by disease. In addition, the effect of EA on different people is affected by many factors, such as smoking, obesity, age and diabetes. Thus, when treating different diseases, the duration and mechanisms of EA are also different.

Undoubtedly, neuroinflammation induced by T lymphocytes is closely related to ischemic stroke. Infiltrating T lymphocytes increased in the brain after stroke within 24 h and peaked around day 3 in other studies [53,54]. The imbalance between Th1 and Th2 appears to be a key factor in stroke. Increased IL-17 levels and fewer Treg numbers are directly associated with stroke onset [55,56]. In the present study, iPSC-EVs and EA treatment modulated the inflammatory response by promoting Treg response and suppressing Th17 and Th1 responses.

IL-33 exerts pleiotropic roles in regulating inflammatory responses in different disease conditions, such as pulmonary sarcoidosis [57] and spinal cord injury [58]. Moreover, numerous studies pointed out that the IL-33/ST2 axis suppresses neuroinflammation in the brain by promoting the Th1/Th2 balance toward Th2 response and inhibiting the Th17 response [28,59]. Activation of IL-33/ST2 leads to polarization of microglia toward the M2 phenotype [43]. A recent study also documented that the CNS protection provided by IL-33 is primarily due to its ability to induce IL-10 production in microglia, whereas genetic deletion of ST2 expanded cerebral infarction by switching microglia toward an M1-like phenotype [29]. Genetic perturbation of IL-33 causes microglial impairment, synaptic dysfunction, and behavioral deficits by damaging the microglia–astrocyte circuit [60].

Our research shows that IL-33/ST2 protein levels are elevated after ischemia, accompanied by astrocyte and microglia activation, and this trend was reduced by both iPSC-EVs and EA treatment, whereas a more significant decrease in these levels was observed in the EA+iPSC-EVs group. We also found that IL-33 is mainly expressed in astrocytes as previously mentioned [61]. The findings presented here may elucidate that EA promotes the protective effect of iPSC-EVs on MCAO mice by modulating the neuroinflammation mediated by the IL-33/ST2 axis. However, the detailed mechanism of iPSC-EVs and EA on the IL-33/ST2 axis requires further exploration. Furthermore, this research provides a novel therapeutic approach to apply EA and iPSC-EVs to clinical ischemic stroke therapy. Nonetheless, the low extraction rate of iPSC-EVs is still a critical challenge in terms of clinical-scale production.

## 5. Conclusions

EA and iPSC-EVs treatments could reduce the infarct volume, improved motor function and neurological performance, and inhibited neuronal apoptosis in ischemic stroke mice. Our findings reveal a synergistic neuroprotective effect of iPSC-EVs and EA on ischemic brain injury by regulating the IL-33/ST2-mediated inflammation and the activation of microglia and astrocytes. This study proposes a novel therapeutic approach for ischemic brain damage and provides a research basis for the application of a combination of EA and iPSC-EVs to treat different types of tissue damage.

## Data Availability

The data that support the findings of this study are available from the corresponding authors upon reasonable request.

## Supplementary Materials

The following supporting information can be downloaded at: https://www.mdpi.com/article/10.3390/cells11050820/s1, Figure S1: All TTC stained images in each group.

## References

[1] Wu S., Wu B., Liu M., Chen Z., Wang W., Anderson C.S., Sandercock P., Wang Y., Huang Y., Cui L. (2019). Stroke in China: Advances and challenges in epidemiology, prevention, and management. Lancet Neurol..

[2] Zhou M., Wang H., Zeng X., Yin P., Zhu J., Chen W., Li X., Wang L., Wang L., Liu Y. (2019). Mortality, morbidity, and risk factors in China and its provinces, 1990–2017: A systematic analysis for the Global Burden of Disease Study 2017. Lancet.

[3] Wang W., Jiang B., Sun H., Ru X., Sun D., Wang L., Wang L., Jiang Y., Li Y., Wang Y. (2017). Prevalence, incidence, and mortality of stroke in China: Results from a nationwide population-based survey of 480,687 adults. Circulation.

[4] Jayaraj R.L., Azimullah S., Beiram R., Jalal F.Y., Rosenberg G.A. (2019). Neuroinflammation: Friend and foe for ischemic stroke. J. Neuroinflamm..

[5] Stroke Unit Trialists’ Collaboration (1997). Collaborative systematic review of the randomised trials of organised inpatient (stroke unit) care after stroke. BMJ.

[6] Shi K., Zou M., Jia D.-M., Shi S., Yang X., Liu Q., Dong J.-F., Sheth K.N., Wang X., Shi F.-D. (2021). tPA mobilizes immune cells that exacerbate hemorrhagic transformation in stroke. Circ. Res..

[7] Li Y., Zhu Z., Lu B., Huang T., Zhang Y., Zhou N., Xuan W., Chen Z., Wen D., Yu W. (2019). Rosiglitazone ameliorates tissue plasminogen activator-induced brain hemorrhage after stroke. CNS Neurosci. Ther..

[8] Henninger N., Fisher M. (2016). Extending the time window for endovascular and pharmacological reperfusion. Transl. Stroke Res..

[9] Emberson J., Lees K.R., Lyden P., Blackwell L., Albers G., Bluhmki E., Brott T., Cohen G., Davis S., Donnan G. (2014). Effect of treatment delay, age, and stroke severity on the effects of intravenous thrombolysis with alteplase for acute ischaemic stroke: A meta-analysis of individual patient data from randomised trials. Lancet.

[10] Saver J.L., Goyal M., Van Der Lugt A., Menon B.K., Majoie C.B.L.M., Dippel D.W., Campbell B.C., Nogueira R.G., Demchuk A.M., Tomasello A. (2016). Time to treatment with endovascular thrombectomy and outcomes from ischemic stroke: A meta-analysis. JAMA.

[11] Brait V.H., Arumugam T.V., Drummond G.R., Sobey C.G. (2012). Importance of T lymphocytes in brain injury, immunodeficiency, and recovery after cerebral ischemia. J. Cereb. Blood Flow Metab..

[12] Dou Z., Rong X., Zhao E., Zhang L., Lv Y. (2019). Neuroprotection of resveratrol against focal cerebral ischemia/reperfusion injury in mice through a mechanism targeting gut-brain axis. Cell. Mol. Neurobiol..

[13] Dolati S., Ahmadi M., Khalili M., Taheraghdam A.A., Siahmansouri H., Babaloo Z., Aghebati-Maleki L., Jadidi-Niaragh F., Younesi V., Yousefi M. (2018). Peripheral Th17/Treg imbalance in elderly patients with ischemic stroke. Neurol. Sci..

[14] Shi L., Sun Z., Su W., Xu F., Xie D., Zhang Q., Dai X., Iyer K., Hitchens T.K., Foley L.M. (2021). Treg cell-derived osteopontin promotes microglia-mediated white matter repair after ischemic stroke. Immunity.

[15] Ito M., Komai K., Mise-Omata S., Iizuka-Koga M., Noguchi Y., Kondo T., Sakai R., Matsuo K., Nakayama T., Yoshie O. (2019). Brain regulatory T cells suppress astrogliosis and potentiate neurological recovery. Nature.

[16] Schmitz J., Owyang A., Oldham E., Song Y., Murphy E., McClanahan T.K., Zurawski G., Moshrefi M., Qin J., Li X. (2005). IL-33, an interleukin-1-like cytokine that signals via the IL-1 receptor-related protein ST2 and induces T helper type 2-associated cytokines. Immunity.

[17] Haraldsen G., Balogh J., Pollheimer J., Sponheim J., Küchler A.M. (2009). Interleukin-33–cytokine of dual function or novel alarmin?. Trends Immunol..

[18] Peine M., Marek R.M., Löhning M. (2016). IL-33 in T cell differentiation, function, and immune homeostasis. Trends Immunol..

[19] Saresella M., Marventano I., Piancone F., La Rosa F., Galimberti D., Fenoglio C., Scarpini E., Clerici M. (2020). IL-33 and its decoy sST2 in patients with Alzheimer’s disease and mild cognitive impairment. J. Neuroinflamm..

[20] Fairlie-Clarke K., Barbour M., Wilson C., Hridi S.U., Allan D., Jiang H.-R. (2018). Expression and function of IL-33/ST2 axis in the central nervous system under normal and diseased conditions. Front. Immunol..

[21] Lamkanfi M., Dixit V.M. (2009). IL-33 raises alarm. Immunity.

[22] Allinne J., Scott G., Lim W.K., Birchard D., Erjefält J.S., Sandén C., Ben L.-H., Agrawal A., Kaur N., Kim J.H. (2019). IL-33 blockade affects mediators of persistence and exacerbation in a model of chronic airway inflammation. J. Allergy Clin. Immunol..

[23] Magat J.M., Thomas J.L., Dumouchel J.P., Murray F., Li W.X., Li J. (2020). Endogenous IL-33 and its autoamplification of IL-33/ST2 pathway play an important role in asthma. J. Immunol..

[24] Miller A.M., Liew F.Y. (2011). The IL-33/ST2 pathway—A new therapeutic target in cardiovascular disease. Pharmacol. Ther..

[25] Pastorelli L., Garg R.R., Hoang S.B., Spina L., Mattioli B., Scarpa M., Fiocchi C., Vecchi M., Pizarro T.T. (2010). Epithelial-derived IL-33 and its receptor ST2 are dysregulated in ulcerative colitis and in experimental Th1/Th2 driven enteritis. Proc. Natl. Acad. Sci. USA.

[26] Sedhom M.A.K., Pichery M., Murdoch J.R., Foligné B., Ortega N., Normand S., Mertz K., Sanmugalingam D., Brault L., Grandjean T. (2013). Neutralisation of the interleukin-33/ST2 pathway ameliorates experimental colitis through enhancement of mucosal healing in mice. Gut.

[27] Qian L., Yuanshao L., Wensi H., Yulei Z., Xiaoli C., Brian W., Wanli Z., Zhengyi C., Jie X., Wenhui Z. (2016). Serum IL-33 is a novel diagnostic and prognostic biomarker in acute ischemic stroke. Aging Dis..

[28] Luo Y., Zhou Y., Xiao W., Liang Z., Dai J., Weng X., Wu X. (2015). Interleukin-33 ameliorates ischemic brain injury in experimental stroke through promoting Th2 response and suppressing Th17 response. Brain Res..

[29] Yang Y., Liu H., Zhang H., Ye Q., Wang J., Yang B., Mao L., Zhu W., Leak R., Xiao B. (2017). ST2/IL-33-dependent microglial response limits acute ischemic brain injury. J. Neurosci..

[30] Takahashi K., Yamanaka S. (2006). Induction of pluripotent stem cells from mouse embryonic and adult fibroblast cultures by defined factors. Cell.

[31] Santoso M.R., Ikeda G., Tada Y., Jung J., Vaskova E., Sierra R.G., Gati C., Goldstone A.B., Von Bornstaedt D., Shukla P. (2020). Exosomes from induced pluripotent stem cell-derived cardiomyocytes promote autophagy for myocardial repair. J. Am. Heart Assoc..

[32] Kim S., Lee S.K., Kim H., Kim T.M. (2018). Exosomes secreted from induced pluripotent stem cell-derived mesenchymal stem cells accelerate skin cell proliferation. Int. J. Mol. Sci..

[33] Oh M., Lee J., Kim Y.J., Rhee W.J., Park J.H. (2018). Exosomes derived from human induced pluripotent stem cells ameliorate the aging of skin fibroblasts. Int. J. Mol. Sci..

[34] Zhang Z.G., Buller B., Chopp M. (2019). Exosomes—Beyond stem cells for restorative therapy in stroke and neurological injury. Nat. Rev. Neurol..

[35] Tian T., Zhang H.-X., He C.-P., Fan S., Zhu Y.-L., Qi C., Huang N.-P., Xiao Z.-D., Lu Z.-H., Tannous B.A. (2018). Surface functionalized exosomes as targeted drug delivery vehicles for cerebral ischemia therapy. Biomaterials.

[36] Song Y., Li Z., He T., Qu M., Jiang L., Li W., Shi X., Pan J., Zhang L., Wang Y. (2019). M2 microglia-derived exosomes protect the mouse brain from ischemia-reperfusion injury via exosomal miR-124. Theranostics.

[37] Xin H., Katakowski M., Wang F., Qian J.-Y., Liu X.S., Ali M.M., Buller B., Zhang Z.G., Chopp M. (2017). MicroRNA-17–92 cluster in exosomes enhance neuroplasticity and functional recovery after stroke in rats. Stroke.

[38] Zhang H., Wu J., Wu J., Fan Q., Zhou J., Wu J., Liu S., Zang J., Ye J., Xiao M. (2019). Exosome-mediated targeted delivery of miR-210 for angiogenic therapy after cerebral ischemia in mice. J. Nanobiotechnol..

[39] Sharma P., Mesci P., Carromeu C., McClatchy D.R., Schiapparelli L., Yates J.R., Muotri A.R., Cline H.T. (2019). Exosomes regulate neurogenesis and circuit assembly. Proc. Natl. Acad. Sci. USA.

[40] Wang H., Chen S., Zhang Y., Xu H., Sun H. (2019). Electroacupuncture ameliorates neuronal injury by Pink1/Parkin-mediated mitophagy clearance in cerebral ischemia-reperfusion. Nitric Oxide.

[41] Chen S., Wang H., Xu H., Zhang Y., Sun H. (2020). Electroacupuncture promotes axonal regrowth by attenuating the myelin-associated inhibitors-induced RhoA/ROCK pathway in cerebral ischemia/reperfusion rats. Brain Res..

[42] Liu S., Mahairaki V., Bai H., Ding Z., Li J., Witwer K.W., Cheng L. (2019). Highly purified human extracellular vesicles produced by stem cells alleviate aging cellular phenotypes of senescent human cells. Stem Cells.

[43] Jiang M., Liu X., Zhang D., Wang Y., Hu X., Xu F., Jin M., Cao F., Xu L. (2018). Celastrol treatment protects against acute ischemic stroke-induced brain injury by promoting an IL-33/ST2 axis-mediated microglia/macrophage M2 polarization. J. Neuroinflamm..

[44] Ziebell J.M., Adelson P.D., Lifshitz J. (2015). Microglia: Dismantling and rebuilding circuits after acute neurological injury. Metab. Brain Dis..

[45] Xu A.-L., Zheng G.-Y., Ye H.-Y., Chen X.-D., Jiang Q. (2020). Characterization of astrocytes and microglial cells in the hippocampal CA1 region after transient focal cerebral ischemia in rats treated with Ilexonin A. Neural Regen. Res..

[46] Turovsky E.A., Varlamova E.G., Plotnikov E.Y. (2021). Mechanisms underlying the protective effect of the peroxiredoxin-6 are mediated via the protection of astrocytes during ischemia/reoxygenation. Int. J. Mol. Sci..

[47] Ye M., Ni Q., Qi H., Qian X., Chen J., Guo X., Li M., Zhao Y., Xue G., Deng H. (2019). Exosomes derived from human induced pluripotent stem cells-endothelia cells promotes postnatal angiogenesis in mice bearing ischemic limbs. Int. J. Biol. Sci..

[48] Joladarashi D., Garikipati V.N.S., Thandavarayan R.A., Verma S.K., Mackie A.R., Khan M., Gumpert A.M., Bhimaraj A., Youker K.A., Uribe C. (2015). Enhanced cardiac regenerative ability of stem cells after ischemia-reperfusion injury: Role of human CD34+ cells deficient in microRNA-377. J. Am. Coll. Cardiol..

[49] Nong K., Wang W., Niu X., Hu B., Ma C., Bai Y., Wu B., Wang Y., Ai K. (2016). Hepatoprotective effect of exosomes from human-induced pluripotent stem cell–derived mesenchymal stromal cells against hepatic ischemia-reperfusion injury in rats. Cytotherapy.

[50] Zhang Y.-M., Xu H., Chen S.-H., Sun H. (2021). Electroacupuncture regulates endoplasmic reticulum stress and ameliorates neuronal injury in rats with acute ischemic stroke. Evid.-Based Complement. Altern. Med..

[51] Zhang Y.-M., Xu H., Sun H., Chen S.-H., Wang F.-M. (2014). Electroacupuncture treatment improves neurological function associated with regulation of tight junction proteins in rats with cerebral ischemia reperfusion injury. Evid.-Based Complement. Altern. Med..

[52] Nierhaus T., Pach D., Huang W., Long X., Napadow V., Roll S., Liang F., Pleger B., Villringer A., Witt D.M.C.M. (2015). Differential cerebral response to somatosensory stimulation of an acupuncture point vs. two non-acupuncture points measured with EEG and fMRI. Front. Hum. Neurosci..

[53] Schroeter M., Jander S., Witte O.W., Stoll G. (1994). Local immune responses in the rat cerebral cortex after middle cerebral artery occlusion. J. Neuroimmunol..

[54] Jander S., Kraemer M., Schroeter M., Witte O.W., Stoll G. (1995). Lymphocytic infiltration and expression of intercellular adhesion molecule-1 in photochemically induced ischemia of the rat cortex. J. Cereb. Blood Flow Metab..

[55] Zhang Q., Liao Y., Liu Z., Dai Y., Li Y., Li Y., Tang Y. (2021). Interleukin-17 and ischaemic stroke. Immunology.

[56] Machhi J., Kevadiya B.D., Khattak M.I.K., Herskovitz J., Olson K.E., Mosley R.L., Gendelman H.E. (2020). Harnessing regulatory T cell neuroprotective activities for treatment of neurodegenerative disorders. Mol. Neurodegener..

[57] Zhang B., Zhao F., Mao H., Ma W., Zhang Y., Zhang X., Ding J., Gao Q., Wen Y. (2018). Interleukin 33 ameliorates disturbance of regulatory T cells in pulmonary sarcoidosis. Int. Immunopharmacol..

[58] Pomeshchik Y., Kidin I., Korhonen P., Savchenko E., Jaronen M., Lehtonen S., Wojciechowski S., Kanninen K., Koistinaho J., Malm T. (2015). Interleukin-33 treatment reduces secondary injury and improves functional recovery after contusion spinal cord injury. Brain Behav. Immun..

[59] Jiang H.-R., Milovanović M., Allan D., Niedbala W., Besnard A.-G., Fukada S.Y., Alves-Filho J.C., Togbe D., Goodyear C.S., Linington C. (2012). IL-33 attenuates EAE by suppressing IL-17 and IFN-γ production and inducing alternatively activated macrophages. Eur. J. Immunol..

[60] He D., Xu H., Zhang H., Tang R., Lan Y., Xing R., Li S., Christian E., Hou Y., Lorello P. (2022). Disruption of the IL-33-ST2-AKT signaling axis impairs neurodevelopment by inhibiting microglial metabolic adaptation and phagocytic function. Immunity.

[61] Korhonen P., Kanninen K.M., Lehtonen S., Lemarchant S., Puttonen K., Oksanen M., Dhungana H., Loppi S., Pollari E., Wojciechowski S. (2015). Immunomodulation by interleukin-33 is protective in stroke through modulation of inflammation. Brain Behav. Immun..

