# Correction: Deng et al. Therapeutic Potential of a Combination of Electroacupuncture and Human iPSC-Derived Small Extracellular Vesicles for Ischemic Stroke. *Cells* 2022, *11*, 820

**DOI:** 10.3390/cells13121015

**Published:** 2024-06-11

**Authors:** Peiying Deng, Liang Wang, Qiongqiong Zhang, Suhui Chen, Yamin Zhang, Hong Xu, Hui Chen, Yi Xu, Wei He, Jianmin Zhang, Hua Sun

**Affiliations:** 1 Department of Traditional Chinese Medicine, Peking Union Medical College Hospital, Peking Union Medical College, Chinese Academy of Medical Sciences, Beijing 100730, China; 2 CAMS Key Laboratory for T Cell and Immunotherapy, State Key Laboratory of Medical Molecular Biology, Department of Immunology, Institute of Basic Medical Sciences, Chinese Academy of Medical Sciences and School of Basic Medicine, Peking Union Medical College, Beijing 100005, China; 3 Changzhou Xitaihu Institute for Frontier Technology of Cell Therapy, Changzhou 213000, China; 4 Guidon Pharmaceutics, Beijing 100176, China

The authors wish to make the following changes to their paper [1]. Due to an error, the following groups in Figure 2F are duplicated: the sham group and the EA + iPSC-EVs group; they therefore need to be corrected. Figure 2 should be changed as follows:

The authors state that the scientific conclusions are unaffected. This correction was approved by the Academic Editor. The original publication has also been updated.

## Figures and Tables

**Figure 2:**
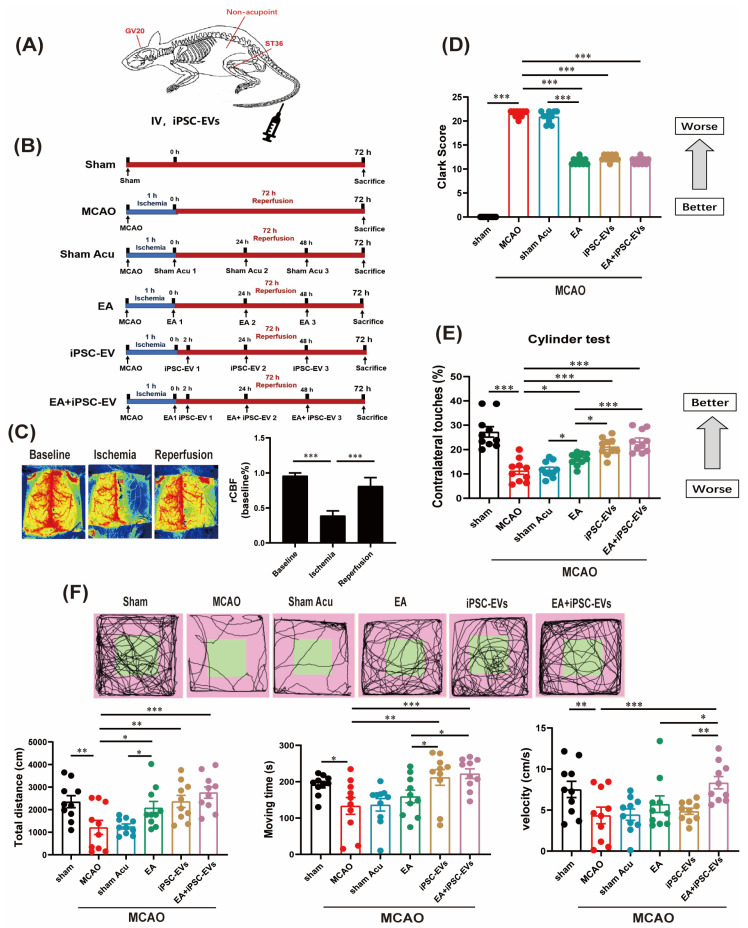
EA stimulation combined with iPSC-EVs improves motor function after ischemic stroke. (**A**) Map of acupoint and nonacupoint locations in mice. (**B**) Timeline of the experimental design in different groups. (**C**) Quantification of rCBF monitored using laser speckle imaging before and after MCAO, as well as 5 min after reperfusion. (**D**) Neurological deficits were evaluated by calculating the Clark score. (**E**,**F**) Cylinder test and open field test were used to assess the deficits in motor function of MCAO mice. Data are shown as means ± SEM. * *p* < 0.05, ** *p* < 0.01, and *** *p* < 0.001.
