# Supplementary figures and images for: Therapeutic Potential of a Combination of Electroacupuncture and Human iPSC-Derived Small Extracellular Vesicles for Ischemic Stroke
Source: Cells. 2022 Feb 26;11(5):820. doi: 10.3390/cells11050820 (PMC8909871; doi:10.3390/cells11050820)

MCAO

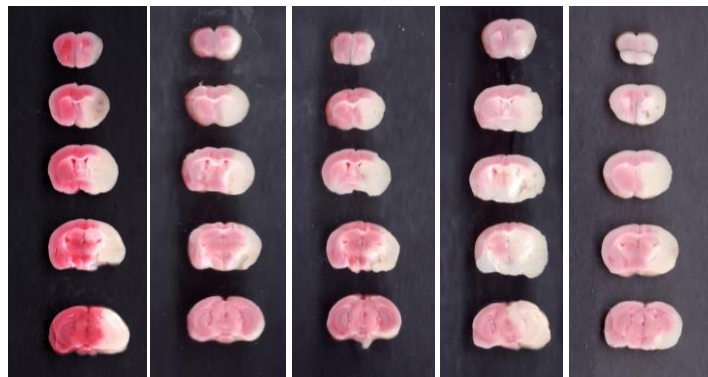

Sham Acu

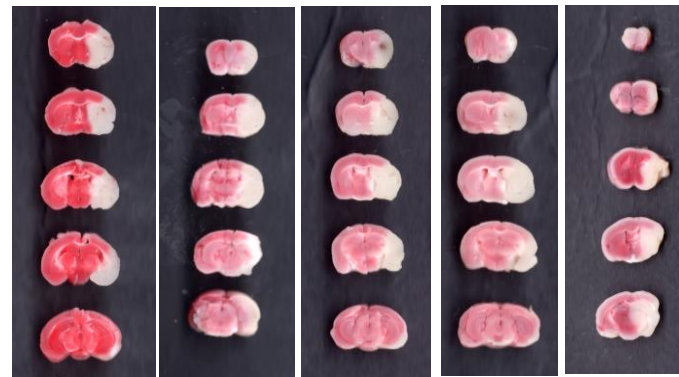

EA

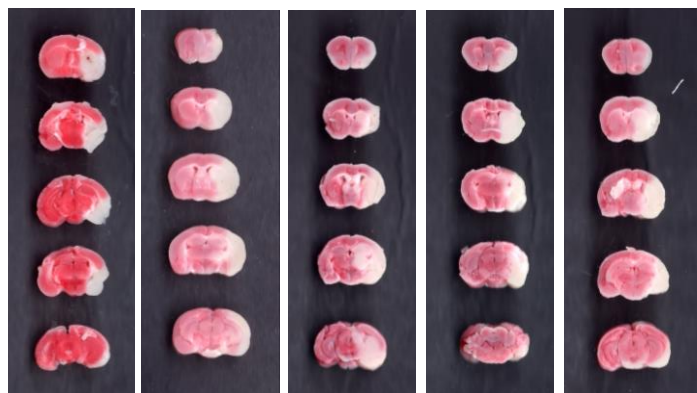

iPSC-EVs

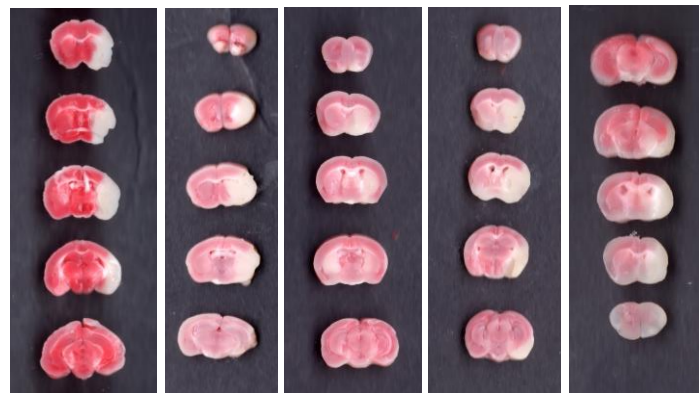

EA+iPSC-EVs

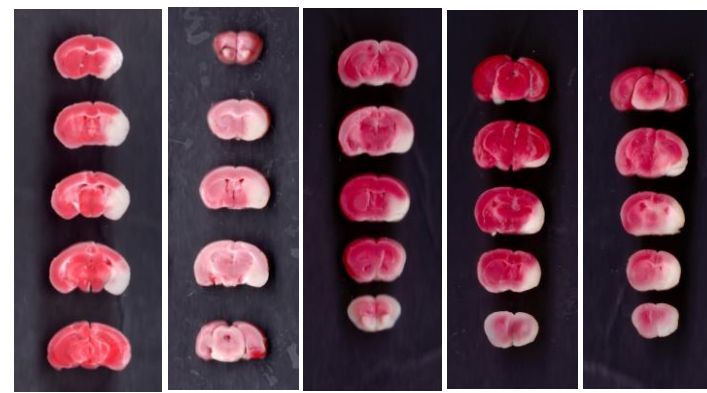

Figure S1. TTC stainings of each group.

Supplement: Supplementary file 1 [file cells-11-00820-s001.zip › cells-1519646-supplementary.pdf]
